# Early Versus Delayed Endovascular Revascularization: Implications for Limb Salvage and Survival After Transmetatarsal Amputation

**DOI:** 10.1016/j.jscai.2026.105623

**Published:** 2026-08-27

**Authors:** Ashkan Yahyavi, Leen Hussein, Zakarias H. Shishehbor, Yara Shatnawi, Maedeh Zokaei Nikoo, Mehdi H. Shishehbor

**Affiliations:** a Department of Cardiovascular Medicine, University Hospitals Harrington Heart and Vascular Institute, Cleveland, Ohio; b Hawken School, Cleveland, Ohio; c Department of Medicine, University Hospitals, Cleveland, Ohio

**Keywords:** amputation, chronic limb-threatening ischemia, endovascular procedures, peripheral arterial disease, revascularization

## Abstract

**Background:**

Healing after transmetatarsal amputation (TMA) depends on adequate tissue perfusion. Revascularization prior to minor amputation improves amputation-free survival, limb preservation, and wound healing in patients with ischemic wounds. However, the optimal interval between revascularization and amputation remains unclear.

**Methods:**

The TriNetX real-world clinical data platform was used to identify patients with chronic limb-threatening ischemia (CLTI) who underwent endovascular revascularization followed by TMA. Patients were categorized into early TMA (≤7 days after revascularization) and late TMA (8-30 days). Propensity score matching was performed using demographics, comorbidities, laboratory values, and concurrent therapies. Major amputation and all-cause mortality at 6 months and 1 year were evaluated using Cox proportional hazards models. Kaplan–Meier curves and log-rank tests were used for time-to-event analyses.

**Results:**

Among 700 patients with CLTI treated with revascularization before TMA, 508 underwent early TMA and 192 underwent late TMA. After matching, 185 patients remained in each group (96.3% match rate). The mean age was 66.9 ± 10.9 years, 78.1% were male, and mean follow-up was 237.2 days. At 6 months, major amputation (hazard ratio [HR], 0.87; 95% CI, 0.51-1.46; *P* =.597) and all-cause mortality (HR, 0.74; 95% CI, 0.45-1.20; *P* = .220) did not differ between groups. At 1-year, major amputation (HR, 0.87; 95% CI, 0.52-1.45; *P* = .599) and mortality (HR, 0.73; 95% CI, 0.46-1.14; *P* = .164) also remained similar. A composite endpoint of major amputation or all-cause mortality was also similar between groups (HR, 0.995; 95% CI, 0.694-1.425; *P* = .978).

**Conclusion:**

Among patients with CLTI undergoing endovascular therapy and subsequent TMA, early (≤7 days) vs late (8-30 days) amputation timing did not significantly affect major amputation or survival outcomes.

## Introduction

Chronic limb-threatening ischemia (CLTI) represents the most advanced stage of peripheral artery disease and is associated with high rates of limb loss, impaired wound healing, and mortality.^1^ Adequate tissue perfusion is essential for successful healing after transmetatarsal amputation (TMA), and prior studies have demonstrated that revascularization before minor amputation improves survival, limb preservation, and wound outcomes.^2^, ^3^, ^4^, ^5^ However, despite the central role of restoring perfusion, the optimal interval between endovascular revascularization and subsequent TMA remains poorly defined. Clinical practice varies widely, influenced by wound characteristics, infection severity, and institutional logistic, yet evidence guiding the timing of amputation within the early postrevascularization period is limited.

This study aimed to determine whether the timing of TMA after endovascular revascularization meaningfully influences major amputation or mortality outcomes in patients with CLTI. Establishing this distinction may help guide more individualized and evidence-based decisions regarding the timing of minor amputation in contemporary limb salvage practice.

## Methods

### Data source and ethical considerations

This retrospective study was performed using the TriNetX database, a global real-world data network that compiles electronic medical records from numerous health care organizations worldwide. The study population was identified from 2011 through 2025 and included data from 39 participating health care organizations. The contributing centers were geographically distributed across the United States, including the Northeast, Midwest, South, and West regions. TriNetX provides advanced tools for cohort creation and data analysis, and the database includes *International Classification of Diseases, 10th Revision* (ICD-10) codes, Current Procedural Terminology (CPT) codes, demographic information, laboratory values, medication records, and clinical outcomes. This study was exempt from institutional review board evaluation because it involved only nonidentifiable data, consistent with the criteria outlined in Section §164.514(a) of the Health Insurance Portability and Accountability Act (HIPAA) Privacy Rule and supported by formal determination from a qualified expert as specified in Section §164.514(b)(1).

### Study population

The study cohorts were identified through 2 distinct data queries. The early TMA group comprised adult patients with a documented history of CLTI, identified through ICD-10 codes, who underwent TMA within 7 days of an endovascular thrombectomy procedure, as determined by the corresponding CPT codes. The late TMA group included adult patients with CLTI who underwent TMA from 8 to 30 days after their revascularization procedure. Laterality was assessed when available; however, procedural CPT data in TriNetX do not consistently confirm that revascularization and TMA occurred in the same extremity. To reduce the likelihood of unrelated contralateral procedures, TMA was required to occur within 30 days after the index revascularization. Details of the patient selection queries are discussed in Supplementary Material.

### Baseline characteristics, propensity score matching, and outcome assessment

Baseline demographic characteristics, laboratory values, and medication records were obtained from the database, and comorbidities were identified using the relevant ICD-10 codes. Propensity score matching was conducted using demographic characteristics, comorbidities, laboratory data, and the use of other antidiabetic medications (Supplementary Figure S1). All variables used for propensity score matching are listed in Table 1.

**Table 1:** Clinical characteristics before and after 1:1 propensity score matching

Characteristic	Before matching			After matching		
	Early TMA (n = 508)	Late TMA (n = 192)	SMD	Early TMA (n = 185)	Late TMA (n = 185)	SMD
Age at index, y	66.5 ± 11.2	66.7 ± 10.6	0.02	67.0 ± 11.1	66.7 ± 10.6	0.02
Male sex	369 (72.6)	144 (75%)	0.05	150 (81.1)	139 (75.1)	0.14
White race	271 (53.3)	98 (51)	0.05	96	98	0.02
Comorbidities						
Diabetes mellitus	457 (90)	172 (89.6)	0.01	169 (91.4)	166 (89.7)	0.06
Chronic kidney disease	284 (55.9)	113 (58.9)	0.06	112 (60.5)	110 (59.4)	0.02
Hypertension	430 (84.6)	163 (84.9)	0.01	160 (86.5)	156 (84.3)	0.06
Hyperlipidemia	333 (65.6)	126 (65.6)	0	128 (69.2)	121 (65.4)	0.08
Ischemic heart disease	279 (54.9)	124 (64.6)	0.2	116 (62.7)	118 (63.8)	0.02
Heart failure	222 (43.7)	86 (44.8)	0.02	77 (41.6)	84 (45.4)	0.08
Obesity	149 (29.3)	61 (31.8)	0.05	54 (29.2)	57 (30.8)	0.04
Tobacco use	142 (28)	59 (30.7)	0.06	51 (27.6)	54 (29.2)	0.04
Medications						
Aspirin	366 (72)	157 (81.8)	0.23	140 (75.7)	150 (81.1)	0.13
Clopidogrel	302 (59.4)	142 (74)	0.31	140 (75.7)	136 (73.5)	0.05
Atorvastatin	327 (64.4)	143 (74.5)	0.22	130 (70.3)	137 (74.1)	0.08
Laboratory values						
Hemoglobin A1c, %	7.9 ± 2.4	7.8 ± 2.2	0.08	7.8 ± 2.2	7.7 ± 2.1	0.08
Serum creatinine, mg/dL	2.5 ± 2.7	2.9 ± 2.9	0.13	2.6 ± 3	2.9 ± 2.9	0.08

Values are mean ± SD or n (%).

SMD, standardized mean difference; TMA, transmetatarsal amputation.

The primary outcome of this study was major (below the knee or above the knee) lower limb amputation identified using relevant CPT codes (Supplementary Table S1), and the secondary outcome was all-cause mortality assessed at 6 months and 1 year after the TMA.

### Statistical analysis

Categorical variables were summarized as percentages and compared using chi-square tests. Continuous variables were reported as mean ± SD and analyzed with independent *t* tests. Propensity score matching was performed based on clinically relevant variables rather than statistical significance. Patients with comparable propensity scores were matched in a 1:1 ratio using a greedy nearest-neighbor algorithm with a caliper of 0.1. Time-to-event analyses were conducted with Kaplan–Meier curves, and differences between groups were assessed using the log-rank test. Cox proportional hazards regression was applied to estimate hazard ratios (HRs) with 95% CIs. Competing-risk analyses were performed using the TriNetX Competing Risks module. Cumulative incidence estimates were calculated using the Aalen–Johansen method with major amputation and all-cause mortality specified as competing events. Statistical significance was defined as *P* < .05. All analyses were performed using the advanced analytics capabilities of the TriNetX online platform.

## Results

### Cohort and patient selection

A total of 700 patients met inclusion criteria prior to matching, of whom 508 underwent early TMA, and 192 underwent late TMA. The dataset included patients from 39 participating health care organizations. Among patients with available regional data, 174 were from the Northeast, 49 from the Midwest, 135 from the South, and 25 from the West. To account for baseline differences between cohorts, propensity score matching was performed using the following variables: demographic, procedural, diagnostic, and laboratory categories. After propensity score matching, 185 patients remained in each cohort, corresponding to a 96.3% match rate for the late TMA group. Baseline variables were well balanced between groups after matching (Table 1).

### Baseline characteristics

The mean age at index was 67.0 ± 11.1 years in the early TMA group and 66.7 ± 10.6 years in the late TMA group. Most patients were male (81.1% vs 75.1%), and the proportion of White patients was similar between cohorts (51.9% vs 53.0%). Both cohorts reflected a high-risk CLTI population with similar comorbidity profiles. Diabetes, hypertension, hyperlipidemia, ischemic heart disease, heart failure, chronic kidney disease, tobacco use, and obesity occurred at comparable rates. Baseline medication use—including aspirin, clopidogrel, and statins—was also well balanced. Laboratory values were similar, and standardized mean differences were uniformly small, confirming excellent covariate balance after matching (Table 1).

### Primary outcome

Six-month major amputation did not differ between groups. Kaplan–Meier estimates showed 82.7% freedom from major amputation in the early TMA cohort and 80.2% in the late TMA cohort (Supplementary Table S3), with no significant difference on log-rank testing (*P* = .597). The HR for major amputation was 0.87 (95% CI, 0.51-1.46), indicating no measurable association between timing of TMA and subsequent amputation risk. One-year major amputation was also comparable between groups. Kaplan–Meier estimates showed 81.5% freedom from major amputation in the early TMA cohort and 79.2% in the late TMA cohort (Supplementary Table S3), with no significant difference on log-rank testing (*P* = .599). The HR for major amputation was 0.87 (95% CI, 0.52-1.45), indicating no measurable association between earlier TMA and subsequent amputation risk at 1 year (Figure 1).

**Figure 1:**
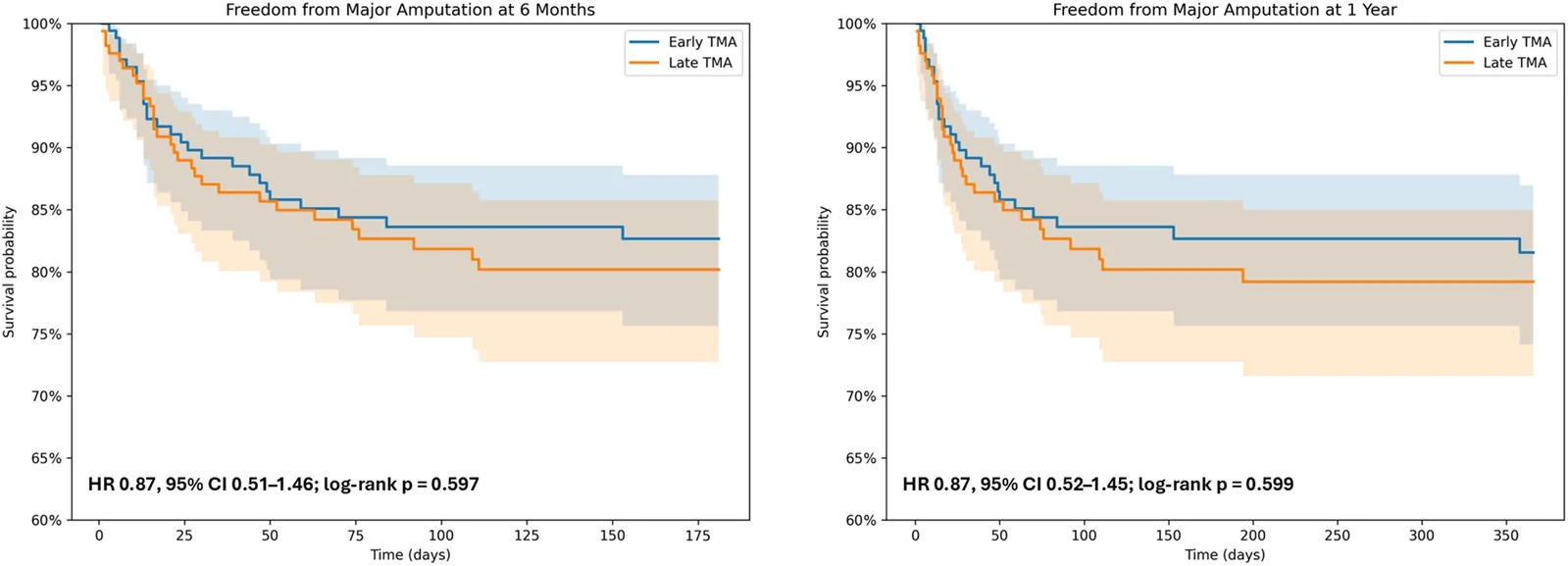
**Kaplan–Meier curves for freedom from major amputation in early vs late TMA at 6 months and 1 year.** HR, hazard ratio; TMA, transmetatarsal amputation.

### Secondary outcomes

Six-month overall survival was similar between groups. Kaplan–Meier estimates showed 82.7% survival in the early TMA cohort and 77.3% in the late TMA cohort (Supplementary Table S2), with no significant difference on log-rank testing (*P* = .220). The HR for all-cause mortality was 0.74 (95% CI, 0.45-1.20), indicating no measurable association between earlier TMA and short-term mortality. One-year overall survival also did not differ between cohorts. Kaplan–Meier estimates demonstrated 79.4% survival in the early TMA group and 72.5% in the late TMA group (Supplementary Table S2), with no significant difference on log-rank testing (*P* = .164). The HR for all-cause mortality was 0.73 (95% CI, 0.46-1.14), again showing no significant association between earlier TMA and mortality risk over 1 year (Figure 2).

**Figure 2:**
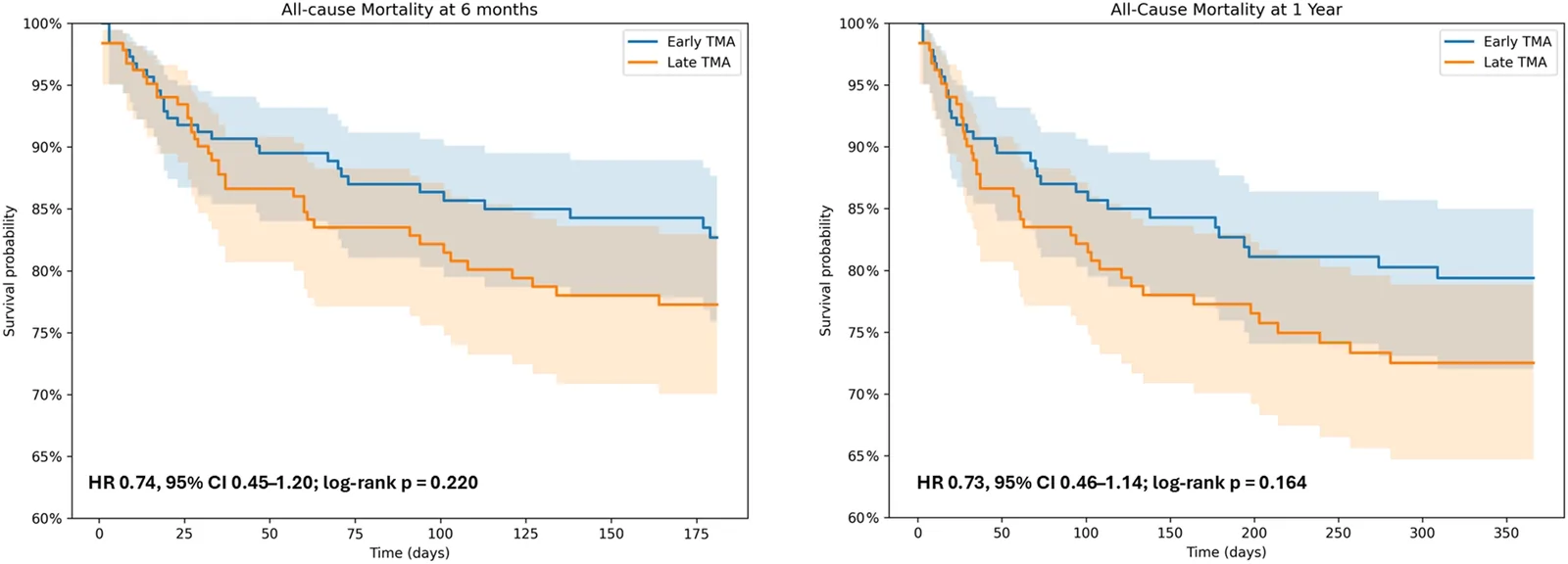
**Kaplan–Meier curves for all-cause mortality inearly vslate TMA at 6 months and 1 year.** HR, hazard ratio; TMA, transmetatarsal amputation.

### Competing risk and composite outcome analyses

Because death represents a clinically relevant competing event for major amputation in patients with CLTI, additional competing-risk analyses were performed. At 6 months, the estimated cumulative incidences of major amputation and death were 17.8% and 17.3%, respectively, in the early TMA cohort and 21.1% and 18.0%, respectively, in the late TMA cohort (Supplementary Table S4). At 1 year, the estimated cumulative incidences of major amputation and death were 18.8% and 21.3%, respectively, in the early TMA cohort and 21.8% and 21.0%, respectively, in the late TMA cohort. These findings demonstrate that major amputation and death occurred at similar frequencies across both cohorts.

To further evaluate whether competing mortality influenced interpretation of the primary outcome, a composite endpoint of major amputation or all-cause mortality was assessed. No significant difference was observed between Early and late TMA groups (HR, 0.995; 95% CI, 0.694-1.425; *P* = .978).

## Discussion

In this multicenter, propensity-matched study of patients with high-risk CLTI undergoing endovascular revascularization and TMA, timing of TMA within 7 days vs 8 to 30 days postrevascularization did not significantly impact major amputation or all-cause mortality at 6 or 12 months (Central Illustration). Results were consistent across analytic methods including competing risk and composite endpoint analyses, indicating that, within 30 days, TMA timing does not independently influence limb salvage or survival. These findings align with recent CLTI literature suggesting that once adequate perfusion is restored, limb outcomes appear to be driven more by the durability and quality of revascularization rather than the specific interval between revascularization and minor amputation in the first 30 days.^1^

**Central Illustration:**
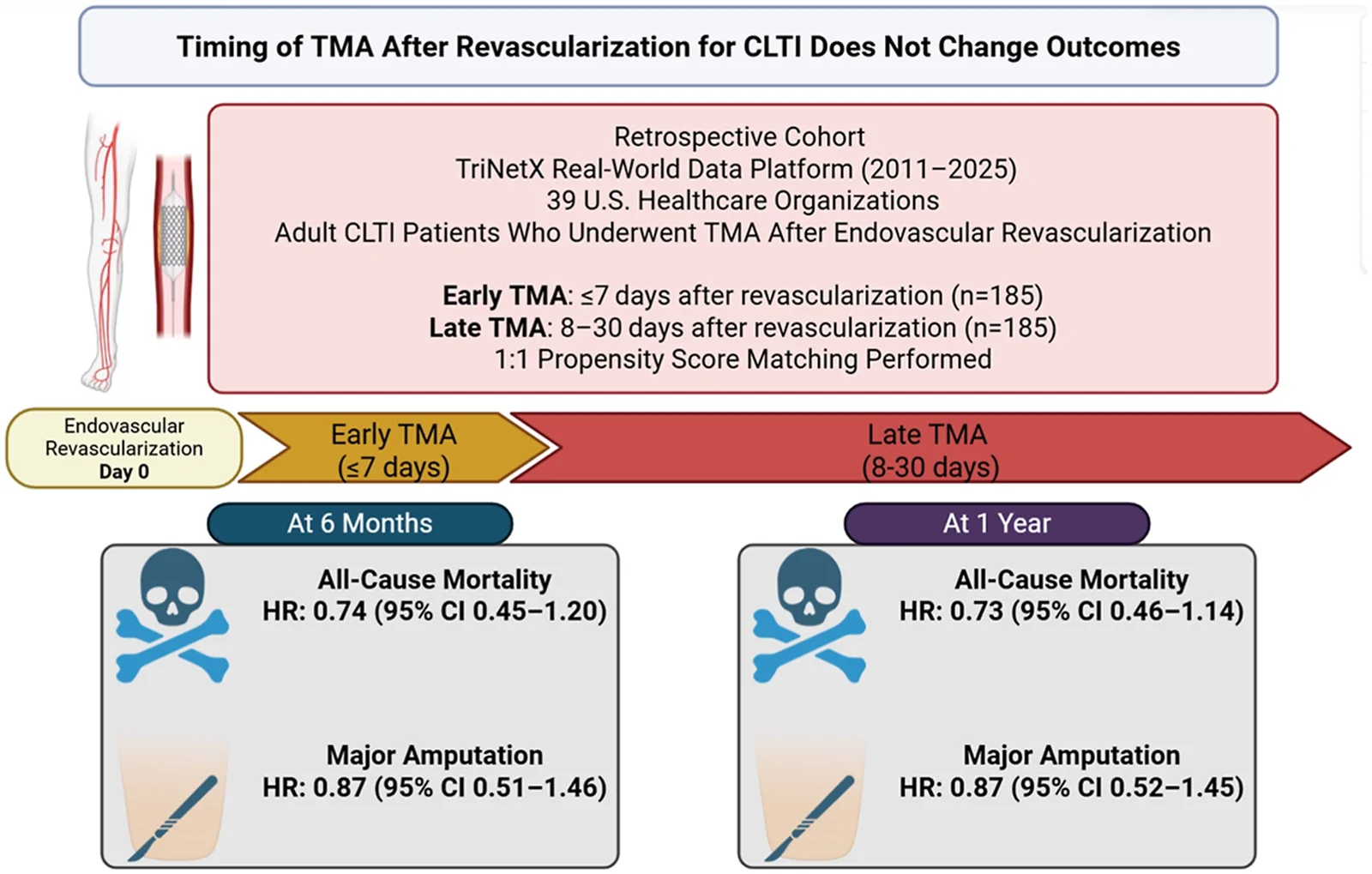
**Summary of study design and outcomes.** CLTI, chronic limb-threatening ischemia; HR, hazard ratio; TMA, transmetatarsal amputation.

CLTI remains a condition with substantial morbidity and mortality. In a large Medicare-based registry analysis of 72,199 patients with newly diagnosed CLTI, Mustapha et al^2^ reported that only 46% of patients survived at 4 years (median survival, 3.5 years), and approximately 29% died or underwent major amputation within the first year. In our matched cohort, 1-year major amputation and mortality rates were consistent with contemporary registry-level data, reinforcing the external validity of our findings.

Prior studies have largely focused on comparing endovascular with surgical revascularization or evaluating outcomes after primary major amputation.^3^ In these studies, long-term survival has generally been comparable between revascularization strategies, whereas both approaches demonstrate superior outcomes compared with primary amputation.^3^

The interval between revascularization and subsequent minor amputation has not been well studied. Chiang et al^4^ demonstrated that patients undergoing revascularization after minor amputation experienced worse outcomes compared with those revascularized beforehand. However, these studies did not specifically evaluate the optimal timing of TMA after successful revascularization. In a recent study, AlRashed et al^1^ categorized minor amputations as same day to ≤7 days or >7 days after revascularization and observed numerically higher future amputation rates across time frames, although the differences between 2 groups were not statistically significant.

Our findings extend these observations by evaluating defined early and late TMAs within a 30-day window and employing propensity matching to reduce confounding, demonstrating that Early vs late TMA within this timeframe does not significantly alter outcomes and highlights the importance of adequate perfusion as the key to improving overall outcomes.

The Global Vascular Guidelines emphasize the importance of staging limb threat using Wound, Ischemia, and foot Infection (WIfI) classification, evaluating anatomic complexity using the Global Limb Anatomic Staging System (GLASS), and selecting revascularization strategies based on patient risk and limb severity. Adequate perfusion is highlighted as being central to wound healing and minor amputation success.^5^ Within TMA-specific outcomes research, multiple studies have shown that TMA healing is heterogeneous and strongly linked to limb perfusion and distal runoff, with downstream failure frequently resulting in higher-level amputation.^6^ Large observational series have highlighted that traditional demographics alone are often insufficient to predict healing, reinforcing the need for physiology- and anatomy-informed approaches.^7^ Pedal arch patency has been shown to associate with improved early healing and limb salvage after TMA, supporting the concept that microvascular and distal conduit factors may matter as much as timing in determining outcomes.^6^^,^^8^

There are several other reasons why timing of TMA within 30 days after endovascular revascularization may not significantly alter risk of major amputation or mortality. First, perfusion benefit may plateau quickly after successful endovascular restoration, such that additional days do not meaningfully change macrovascular inflow once patency is established. The decisive determinants of healing may instead be pedal perfusion, arch completeness, and microvascular function rather than elapsed time alone.^6^ Second, decision for early vs late TMA is mostly a clinical decision, and practice patterns are often driven by wound status, infection control, operating room logistic, and surgeon preference. Patients with severe infection, necrosis, or urgent source control may undergo earlier TMA, whereas those with complex wounds or need for demarcation may be delayed.^9^ With robust propensity matching, the net outcome signal attributable strictly to timing may be minimized, reflecting that clinicians are already individualizing timing appropriately. High comorbidity prevalence (diabetes, chronic kidney disease, coronary artery disease/heart failure) can dominate mortality and limb trajectories, potentially overwhelming modest effects of timing differences within a 30-day window.^9^ This may reduce pressure for “urgent” amputation when not otherwise clinically indicated, while still reinforcing the foundational principle that revascularization adequacy and distal perfusion assessment remain central to limb salvage.^9^ In a study by Doyle et al,^10^ the optimal healing between revascularization and amputation was <30 days, and the authors emphasized that diabetes mellitus and end-stage renal disease were contributing factors for poor healing postrevascularization. Overall, this study reinforces previous findings that limb outcomes are frequently constrained by distal bed perfusion and wound biology, not simply timing of surgical debridement or minor amputation.^11^

### Limitations

Several limitations should be acknowledged. First, this study utilized a retrospective observational design, which may be subject to residual confounding. Second, the analysis depended on the TriNetX federated electronic health record network, which relies on coding accuracy and completeness and does not ensure consistent data granularity across participating health care organizations. Although laterality-specific diagnostic codes were used when available, TriNetX procedural data do not consistently permit confirmation that revascularization and subsequent TMA occurred in the same extremity, introducing the possibility of sidedness misclassification. Similarly, CPT-based identification of TMA did not reliably distinguish open from closed TMA procedures.

TriNetX also lacks granular anatomic, procedural, and hemodynamic data that may influence TMA healing and limb outcomes. Specifically, the database does not provide detailed clinical information regarding GLASS stage, tibial or pedal runoff quality, device strategy, inline flow restoration, target-vessel patency, residual stenosis, or technical success of the index endovascular procedure. In addition, repeat vascular intervention was not assessed in the primary analysis and may influence both the timing of TMA and subsequent limb outcomes. The database also lacks wound-level detail, including WIfI stage, soft tissue extent, infection severity, and osteomyelitis burden. Hemodynamic variables such as ankle-brachial index and toe-brachial index were limited by substantial missingness and uncertain timing relative to revascularization; therefore, objective stratification of ischemia severity was not performed. Because TriNetX relies on prespecified relative time windows, TMA timing could not be analyzed as a continuous variable, and alternative timing thresholds could not be tested.

Although competing-risk analyses were performed, the current TriNetX platform does not support advanced competing-risk comparative analyses between cohorts. Therefore, competing risk estimates were descriptive and were interpreted in conjunction with the primary time-to-event and composite endpoint analyses.

Finally, the outcomes assessed were limited to major amputation and mortality. Important patient-centered endpoints, including time to wound healing, reintervention rates, ambulatory status, quality of life, hospital length of stay, procedure burden, and cost, were not reliably captured in the TriNetX output used for this study.

Future prospective studies with more rigorous design and granular clinical data should include standardized wound staging (WIfI), distal perfusion metrics, inframalleolar anatomy (pedal arch status), and procedural success parameters. These measures will help identify subgroups for whom timing may affect outcomes, such as patients with active infection versus ischemia-predominant wounds or those with adequate versus incomplete revascularization. Assessing both early intervention (same day vs 1-7 days) and longer delays (>30 days) in studies specifically designed to evaluate timing may help define clinically significant timing thresholds.

## Conclusion

In this multicenter propensity-matched study of patients with CLTI undergoing endovascular revascularization followed by TMA, early (≤7 days) vs delayed (8-30 days) TMA was not associated with differences in major amputation or all-cause mortality at 6 or 12 months. These findings suggest that, within 30 days of successful revascularization, TMA timing does not independently influence limb salvage or survival. Adequate and durable perfusion remains the key determinant of outcomes, allowing timing of minor amputation to be individualized based on clinical factors without compromising major limb events.

## CRediT authorship contribution statement

**Ashkan Yahyavi:** Writing – review & editing, Writing – original draft, Visualization, Project administration, Methodology, Formal analysis, Data curation, Conceptualization. **Leen Hussein:** Writing – review & editing, Writing – original draft, Formal analysis, Data curation. **Zakarias H. Shishehbor:** Writing – review & editing, Writing – original draft. **Yara Shatnawi:** Writing – review & editing, Writing – original draft. **Maedeh Zokaei Nikoo:** Writing – review & editing, Writing – original draft. **Mehdi H. Shishehbor:** Writing – review & editing, Supervision, Project administration, Methodology, Conceptualization.

## Declaration of competing interest

Mehdi Shishehbor serves as a global advisor for Boston Scientific, Medtronic, Abbott Vascular, Inquis, ANT, and Inari/Stryker. The remaining authors reported no financial interests.
