# Supplementary material for: Early Versus Delayed Endovascular Revascularization: Implications for Limb Salvage and Survival After Transmetatarsal Amputation
Source: J Soc Cardiovasc Angiogr Interv. 2026 Aug 27;5(10):105623. doi: 10.1016/j.jscai.2026.105623 (PMC13700053; doi:10.1016/j.jscai.2026.105623)
Supplement: Supplementary file 1 [file mmc1.docx]

**Supplementary Material**

Two separate data queries were built to identify the study population.

**Cohort 1: Early TMA Group**

Adults aged ≥18 years with a diagnosis of chronic limb‑threatening ischemia (CLTI), identified using the ICD‑10 codes listed below, who underwent an endovascular revascularization procedure (per CPT codes listed below) and subsequently received a transmetatarsal amputation (TMA) within ≤7 days of the revascularization procedure (per specified CPT code). This group constituted the Early TMA cohort.

**Cohort 2: Late TMA Group**

Adults aged ≥18 years with a diagnosis of CLTI (ICD‑10 codes below) who underwent an endovascular revascularization procedure (CPT codes below) and subsequently received a TMA 8–30 days after the revascularization procedure. This group constituted the Late TMA cohort.

**Supplementary Table S1:** All ICD-10 codes and CPT codes used for the data query.

| ICD-10 code | Explanation |
| --- | --- |
| I70.22 | Atherosclerosis of native arteries of extremities with rest pain |
| I70.23 | Atherosclerosis of native arteries of right leg with ulceration |
| I70.24 | Atherosclerosis of native arteries of left leg with ulceration |
| I70.26 | Atherosclerosis of native arteries of extremities with gangrene |
| I70.32 | Atherosclerosis of unspecified type of bypass graft(s) of the extremities with rest pain |
| I70.33 | Atherosclerosis of unspecified type of bypass graft(s) of the right leg with ulceration |
| I70.34 | Atherosclerosis of unspecified type of bypass graft(s) of the left leg with ulceration |
| I70.36 | Atherosclerosis of unspecified type of bypass graft(s) of the extremities with gangrene |
| I70.42 | Atherosclerosis of autologous vein bypass graft(s) of the extremities with rest pain |
| I70.43 | Atherosclerosis of autologous vein bypass graft(s) of the right leg with ulceration |
| I70.44 | Atherosclerosis of autologous vein bypass graft(s) of the left leg with ulceration |
| I70.46 | Atherosclerosis of autologous vein bypass graft(s) of the extremities with gangrene |
| I70.52 | Atherosclerosis of nonautologous biological bypass graft(s) of the extremities with rest pain |
| I70.53 | Atherosclerosis of nonautologous biological bypass graft(s) of the right leg with ulceration |
| I70.54 | Atherosclerosis of nonautologous biological bypass graft(s) of the left leg with ulceration |
| I70.56 | Atherosclerosis of nonautologous biological bypass graft(s) of the extremities with gangrene |
| I70.62 | Atherosclerosis of nonbiological bypass graft(s) of the extremities with rest pain |
| I70.63 | Atherosclerosis of nonbiological bypass graft(s) of the right leg with ulceration |
| I70.64 | Atherosclerosis of nonbiological bypass graft(s) of the left leg with ulceration |
| I70.66 | Atherosclerosis of nonbiological bypass graft(s) of the extremities with gangrene |
| I70.72 | Atherosclerosis of other type of bypass graft(s) of the extremities with rest pain |
| I70.73 | Atherosclerosis of other type of bypass graft(s) of the right leg with ulceration |
| I70.74 | Atherosclerosis of other type of bypass graft(s) of the left leg with ulceration |
| I70.76 | Atherosclerosis of other type of bypass graft(s) of the extremities with gangrene |
| E11 | Type 2 Diabetes Mellitus |
| I10 | Essential (primary) hypertension |
| I50 | Heart failure |
| I25 | Chronic ischemic heart disease |
| F17 | Nicotine dependence |
| E66 | Overweight and obesity |
| N18 | Chronic kidney disease (CKD) |
| E78.5 | Hyperlipidemia, unspecified |
| CPT Codes | **Explanation** |
| 28805 | Amputation, foot; transmetatarsal |
| 1014590 | Amputation, leg, through tibia and fibula |
| 27880 | Amputation, leg, through tibia and fibula |
| 1005146 | Amputation Procedures on the Femur (Thigh Region) and Knee Joint |
| 1014580 | Amputation, thigh, through femur, any level |
| 27590 | Amputation, thigh, through femur, any level |
| 27882 | Amputation, leg, through tibia and fibula; open, circular (guillotine) |
| 27592 | Amputation, thigh, through femur, any level; open, circular (guillotine) |
| 27594 | Amputation, thigh, through femur, any level; secondary closure or scar revision |
| 27881 | Amputation, leg, through tibia and fibula; with immediate fitting technique including application of first cast |
| 27591 | Amputation, thigh, through femur, any level; immediate fitting technique including first cast |

**Supplementary Figure S1:** Kernel density plots of the propensity scores before and after matching; Purple: Early TMA group, Green: Late TMA Group

**Supplementary Table S2:** Kaplan–Meier Survival Estimates at Prespecified Time Points

| Survival (%) | | | | | |
| --- | --- | --- | --- | --- | --- |
|  | 30 days | 60 days | 120 days | 180 days | 365 days |
| Early TMA | 91.2 | 89.5 | 84.9 | 82.7 | 79.4 |
| Late TMA | 90.1 | 84.8 | 79.4 | 77.3 | 72.5 |

**Supplementary Table S3:** Kaplan–Meier Freedom-From-Major-Amputation Estimates at Prespecified Time Points

| Freedom From Major Amputation (%) | | | | | |
| --- | --- | --- | --- | --- | --- |
|  | 30 days | 60 days | 120 days | 180 days | 365 days |
| Early TMA | 89.2 | 85.1 | 83.6 | 82.7 | 81.5 |
| Late TMA | 87.1 | 84.9 | 80.2 | 80.2 | 79.2 |

TMA, trans-metatarsal amputation

**Supplementary Table S4:** Competing-Risk Cumulative Incidence Estimates

| **Outcome** | **Early TMA 6 mo** | **Late TMA 6 mo** | **Early TMA 1 yr** | **Late TMA 1 yr** |
| --- | --- | --- | --- | --- |
| Major amputation | 17.8% | 21.1% | 18.8% | 21.8% |
| Death | 17.3% | 18.0% | 21.3% | 21.0% |

TMA, trans-metatarsal amputation
